# Early-Age Performance Analysis of Sludge Water Incorporation in High-Temperature Steam Cured Green High-Performance Concrete

**DOI:** 10.3390/ma15051912

**Published:** 2022-03-04

**Authors:** Beimeng Qi

**Affiliations:** College of Quality & Safety Engineering, China Jiliang University, Hangzhou 310018, China; qbm@cjlu.edu.cn

**Keywords:** sludge water, steam curing, high performance concrete, mechanical properties

## Abstract

Sludge water (SW) with abundant sulfate ions (SO_4_^2−^) was utilized in this work to replace freshwater (FW) to prepare green high performance concrete (GHPC). A comprehensive investigation was conducted to evaluate the early-age performance of GHPC specimen mixed with SW incorporation (GHPC-SW). High temperature steam curing (HTS) has been presented to prepare GHPC-SW specimens. The compressive strength of the GHPC-SW specimen cured by HTS curing for 2 days is 85.2 MPa, which is 34% higher than the compressive strength of the GHPC-SW specimen cured by 3 days standard curing as the reference. The mechanical property results reveal that the incorporation of SW makes no harmful effects on the strength formation of HPC specimens, compared with FW added specimens under the same curing methods. Moreover, XRD and TG analyses indicate that HTS curing can effectively improve the hydration degree of GHPC-SW specimens. MIP analysis has been conducted and the specimens cured by HTS curing exhibit a more refined pore structure with fewer harmful pores. This work lays a solid foundation for the utilization of SW in the concrete construction industry, which is resource saving and environmentally friendly.

## 1. Introduction

The concrete construction industry is meeting tremendous development in the last decades and is expected to be more prosperous in the future. However, the rapidly ongoing concrete construction industry has brought a great burden to the environment. Generally speaking, in the process of actual concrete construction, the mass ratio of water to cement is around 0.5, which is very exaggerated for the utilization of rare freshwater resources [1]. Especially in view of the increasing scarcity of freshwater resources in the world, there must be a reasonable way to rationally solve the huge amount of water needed by the growing concrete construction industry. A suitable substitution of freshwater for concrete construction is in great demand to solve this tough problem.

To have the required quality, raw water is treated in water treatment plants before distribution after a complicated process of coagulation-flocculation. The sludge water from the water treatment plants accounts for about 2~4% of the total production [2], which mainly consists of the sludge discharge from the reaction tank, sedimentation tank and backwash water from the filter. There are a large number of suspended impurities, metal ions, organic matter and microorganisms in sludge water. The sludge water discharged into natural water bodies nearby is not conducive to the sustainable development of waterworks and will cause damage to the water environment and water ecology. Large quantities of water treatment sludge, a heterogeneous waste produced daily, and its capitalization are becoming the main concern. Therefore, a proper treatment method is essential for the utilization of sludge water towards environmental protection and resource management.

Cement-based materials possess great potential on immobilizing harmful ions [3,4,5] inside the specimen and the utilization of cement-based materials for disposing of the sludge water obtained from the water production process is a promising way to not only relieve the freshwater crisis but also solve the disposal problem of sludge water due to its highly toxic properties. High-Performance concrete with excellent mechanical properties and good durability has been presented for several decades to achieve the performance-optimization of cement-based materials [6,7]. The achievement of high-Performance concrete is closely related to the utilization of supplementary cementitious materials, the reduction of the water to binder ratio and the implementation of high-temperature curing methods, which are beneficial for enhancing the hydration degree and refining the pore structure of the specimens [8,9], and this is considered to be useful for immobilizing the residue ions inside the sludge water.

Based on the discussion above, this work presents sludge water to fully replace freshwater for preparing high-performance concrete under high-temperature steam curing. The main purpose of this work is to clarify the specific effect of high-temperature steam curing on immobilizing the harmed ions inside the sludge water to improve the performance of the specimen, aiming at utilizing the sludge water as mixing water for cement-based materials with promising performance. The mechanical properties of the specimens with different curing methods and mixing water types were measured to elucidate the effect of sludge water addition on the strength formation of the specimens. Further microscopic analyses (including XRD, TG and MIP) were further conducted to investigate the microstructural evolution regularity of the specimens incorporating sludge water cured with different curing methods. Moreover, the harmful SO_4_^2−^ ion content inside the specimen was also measured to investigate the effect of high-temperature steam curing on the ion binding ratio of the specimen.

## 2. Materials and Methods

### 2.1. Raw Materials

Ordinary Portland cement (OPC) with a strength grade of 42.5 has been utilized as the main cementitious material in this work, detailed composition of the cement is listed in Table 1. Silica fume was used as the supplementary cementitious material in this work. Fine silica sand with an average grain size of 1.5 mm has been served as the fine aggregate. The sludge water (SW) with the main harmful ions of SO_4_^2−^ was used in this work to fully replace freshwater as mixing water.

### 2.2. Preparation of High Performance Concrete Incorporating Sludge Water (HPC-SW)

The water to binder ratio for the HPC-SW specimen was kept as 0.24, and the ratio of binder to sand was determined to be 1:1, the mass ratio of silica fume to cement was 0.2. Moreover, the specific preparation of the HPC-SW specimen is as follows: the mixture of cement and silica fume was initially mixed in the stirring pot at a high speed for 3 min, then the solution of SW and SP was poured into the mixture, followed by 2 min of high-speed stirring. Finally, fine silica sand was put into the pot and the mixture was further mixed with the high speed of 5 min. After the mixing process, the mixture was put to the mold with the size of 40 mm × 40 mm × 160 mm.

Further, the detailed conditions for the utilized curing methods are as below: (1) Standard curing at 20 ± 2 °C (RH > 95%); (2) High temperature steam curing at 90 °C (RH 99%).

### 2.3. Measurement of Mechanical Properties

The mechanical properties of HPC with the incorporation of freshwater (HPC-FW) were measured to investigate the impact of SW on the strength development of GHPC. Specifically, the curing duration of standard curing (SC) was set to be 3 days, and the corresponding duration of high temperature steam (HTS) curing was determined to be 2 days. To promise the accuracy of the tested results, five specimens were prepared for each batch to calculate the average strength. Particularly, the compressive strength measurement loading rate was set to be 2.4 kN/s according to GB/T17671-1999. Moreover, in accordance with GB/T17671-1999, the loading rate of the three-point bending test was determined to be 0.05 kN/s to measure the flexural strengths of the specimens.

### 2.4. Characterization

HPC specimens cured with different conditions were, respectively, cured for SC and HTS curing, and the hydration of the specimens was stopped by immersing the broken specimens into the ethanol, and then the specimens were ground into powder in an agate mortar for microstructural characterizations. To further investigate the influence of curing methods on the microstructure evolution of HPC-SW specimens, the microstructural analyses of HPC-SW specimens cured by different curing methods were also conducted. Specifically, X-ray diffraction (XRD) analysis via X’pert PRO diffractometer (PANalytical B.V., Netherlands)was conducted to figure out component composition phases inside the specimens; 5–55° was set to be the range of the 2-Theta values. Thermal gravity (TG) analysis was conducted to determine the contents of the hydration products of HPC-SW specimens subjected to SC curing and HTS curing. (STA449F3, Netzsch Company, Germany). The heating rate of TG analysis was determined to be 10 °C/min and Nitrogen was determined to be the protective gas with a heating temperature within the range of 30~800 °C under a nitrogen atmosphere. The pore structure distribution of HPC-SW specimens was measured by an IV 9510 Mercury Intrusion Porosimetry (Micromeritics, America) and the pressure was set within the range of 0–60,000 psi. 

## 3. Results

### 3.1. Mechanical Properties

**Figure 1:**
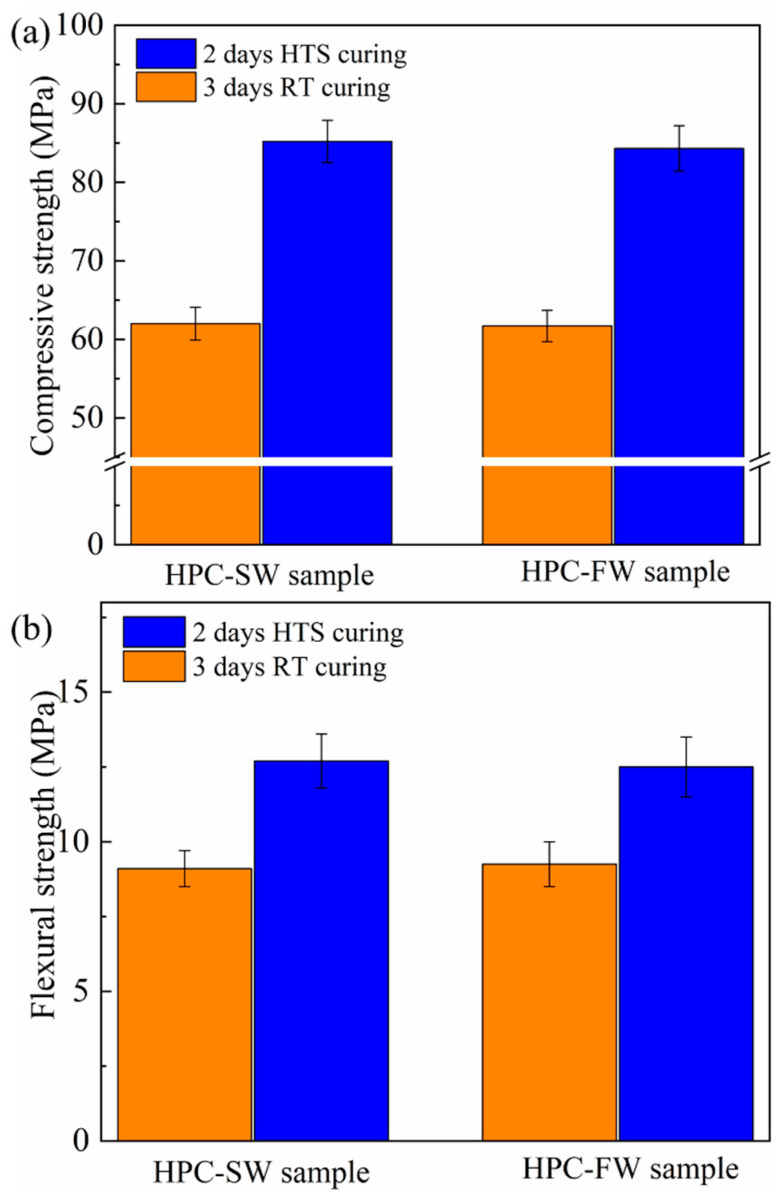
Mechanical properties of HPC-FW and HPC-SW specimens cured by different curing methods: (**a**) compressive strength and (**b**) flexural strength.

The mechanical properties of HPC-FW specimens and HPC-SW specimens cured by different curing methods are shown in Figure 1. It can be seen from Figure 1a that with the incorporation of sludge water, the compressive strength of HPC specimens show little difference, while the compressive strengths between the specimens cured by different curing methods exhibit obvious difference. Specifically, the compressive strength of the HTS cured HPC-SW specimen reaches up to 85.2 MPa, meeting an increase of 34% compared with that of the specimen cured by SC curing with the value of 62 MPa. Moreover, the compressive strength of the HTS cured HPC-FW specimen reaches up to 84.3 MPa, showing little difference with that of the HPC-SW specimen with the same curing method. Furthermore, the compressive strength of the SC cured HPC-FW specimen also shows no statistics error with that of the SC cured HPC-SW specimen. For the flexural strength of the HPC specimens, HTS curing also exhibits the priority to stimulate the strength formation of the HPC specimens. The flexural strength of the HPC-SW specimen cured by HTS curing reaches up to 12.7 MPa, which is comparable with that of the HTS cured HPC-FW specimen with a value of 12.5 MPa. Moreover, the flexural strengths of the HPC-SW and HPC-FW specimens cured by SC curing are 9.1 MPa and 9.25 MPa, respectively. It can be concluded that HTS curing exhibits great potential to stimulate the flexural strength formation of HPC specimens with the incorporation of sludge water.

The mechanical property results indicate that the incorporation of sludge water exhibits no adverse effect on the strength formation of HPC specimens. As reported, HTS curing is an effective curing regime to stimulate the strength development of HPC specimens. Based on the results of mechanical properties, further microstructural analysis is crucial for evaluating the feasibility of the sludge water incorporation of HPC construction.

### 3.2. XRD Analysis

**Figure 2:**
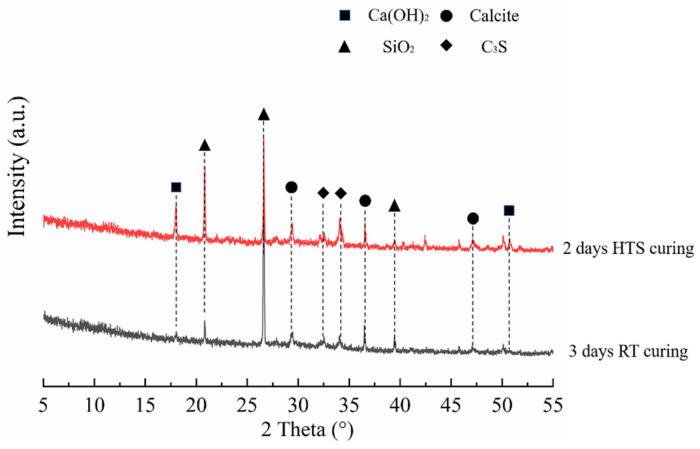
XRD patterns of HPC-SW specimens cured by HTS curing and SC.

XRD analysis was conducted to identify the composition phases of HPC-SW specimens subjected to different curing methods, and the detailed XRD analysis results are shown in Figure 2. It can be seen from Figure 2 that the HPC-SW specimens subjected to different curing regimes exhibit the consistent hydration products system [9]. Moreover, it can be seen from Figure 2 that the intensity of CH inside the HPC-SW specimen cured by HTS curing is much higher than that in the specimen cured by SC, indicating a higher hydration degree of the HTS cured specimen. Furthermore, a great amount of unreacted cement can be observed in the specimens, and this is in accordance with the characteristic of HPC specimens containing lower water content [10,11]. Specifically, the strength of cement-based materials mainly comes from the hydration effect of C_3_S at an early age, because of the dominating role of C_3_S to react with water to produce Ca(OH)_2_. It can be seen from Figure 2 that the HPC-SW specimens cured by SC have a higher C_3_S level, at the same time, the specimen subjected to HTS curing exhibits a much lower content of C_3_S. The lower C_3_S level of the specimens cured by HTS curing further verifies the effect of HTS curing on improving the hydration degree, which is inconsistent with the higher CH content.

### 3.3. TG Analysis

**Figure 3:**
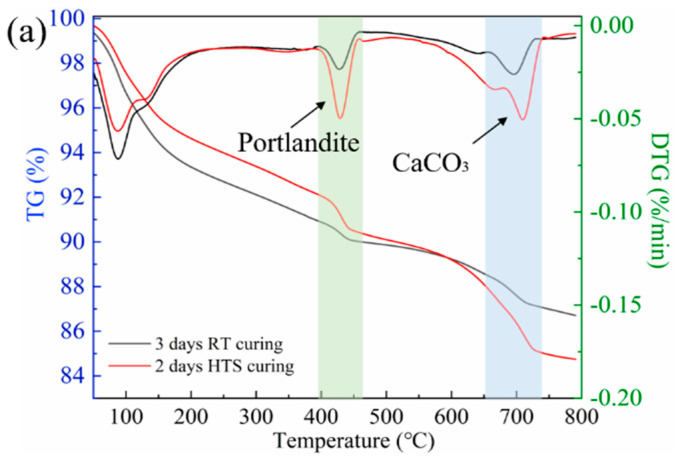

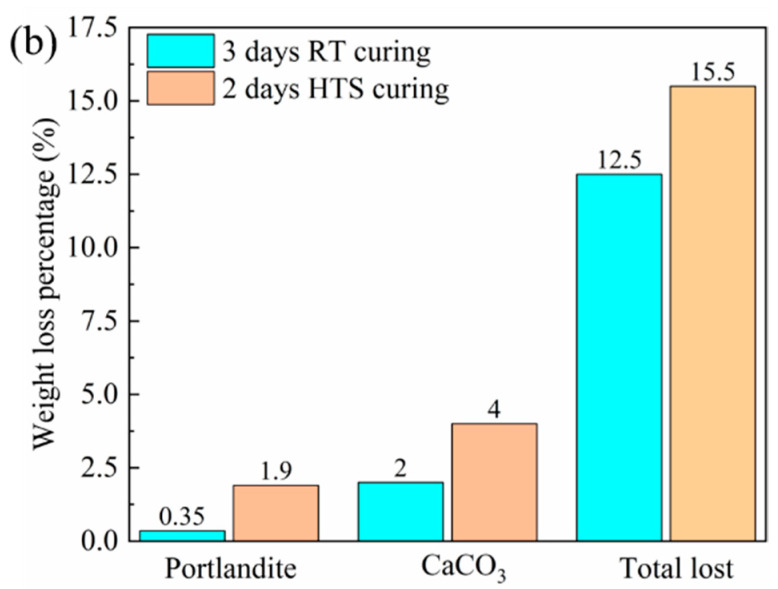
TG analysis of HPC−SW specimens cured by SC curing and HTS curing: (**a**) TG and DTG analyses and (**b**) Weigh loss percentages of Portlandite and CaCO_3_.

Figure 3 shows the TG analysis results of the HPC-SW specimens cured by HTS curing and SC curing. Moreover, the first order differential treatment has been conducted to obtain the differential thermogravigram (DTG) curve of the TG curve and has also been depicted in Figure 3. The DTG curve is a direct exhibition of the heat absorption peaks in accordance with different thermal decomposition processes. Specifically, TG analysis can effectively determine the content of hydration products such as C-S-H gel, ettringite (AFt), crystalline portlandite (CH), and CaCO_3_ [12,13]. As shown in Figure 3, DTG curves show three main heat adsorption peaks. In detail, the first peak shown around 120 °C is mainly caused by the composition of C-S-H gel and AFt. The second peak shown within the range of 400–450 °C represents the decomposition of portlandite. Moreover, the third peak within the scope of 700–750 °C is closely related to CaCO_3_ decomposition. The detailed weight loss of main hydration products (portlandite and CaCO_3_) and the total loss of the specimens cured by different curing methods are listed in Figure 3b. It can be seen from Figure 3b that the HPC-SW specimen cured by HTS curing exhibits higher portlandite content (1.9%) compared with that of the specimen cured by SC (0.35%), indicating a higher hydration degree. Moreover, the higher CaCO_3_ content has also been detected in the HTS cured specimen, emphasizing a stronger pozzolanic reaction. With the higher hydration degree and stronger pozzolanic reaction, HTS curing exhibits great potential to stimulate the micro reaction inside the HPC-SW specimen, further highlighting the priority of HTS curing as an effective curing strategy to prepare HPC specimens with the incorporation of sludge water.

### 3.4. MIP Analysis

**Figure 4:**
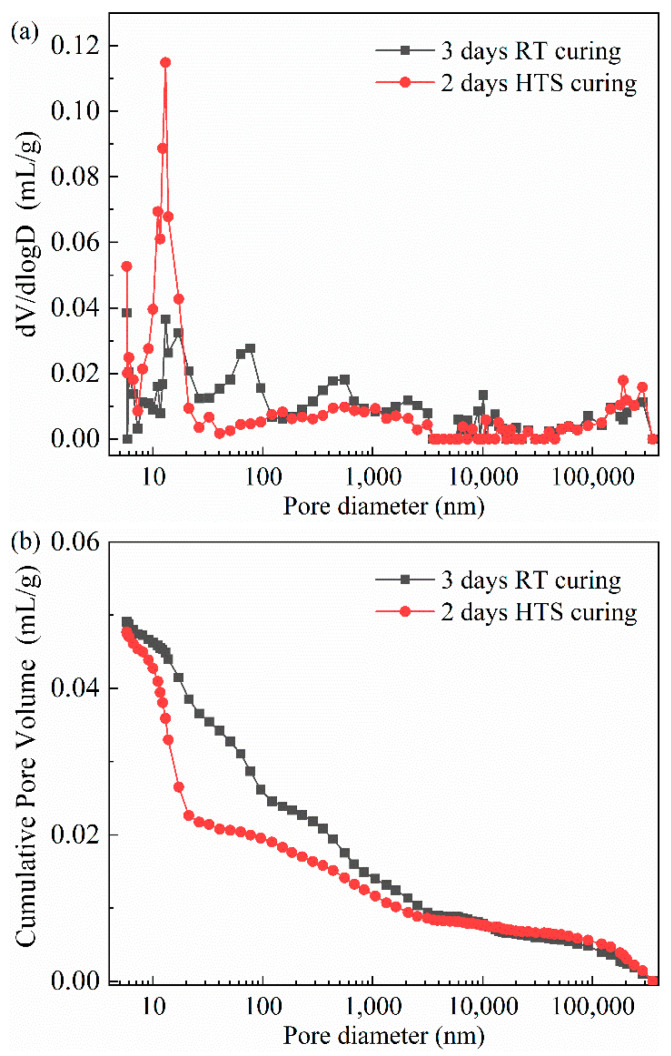
Pore structure analysis of HPC-SW specimens cured by different curing methods: (**a**) distribution of pore size and (**b**) cumulative pore volume.

**Figure 5:**
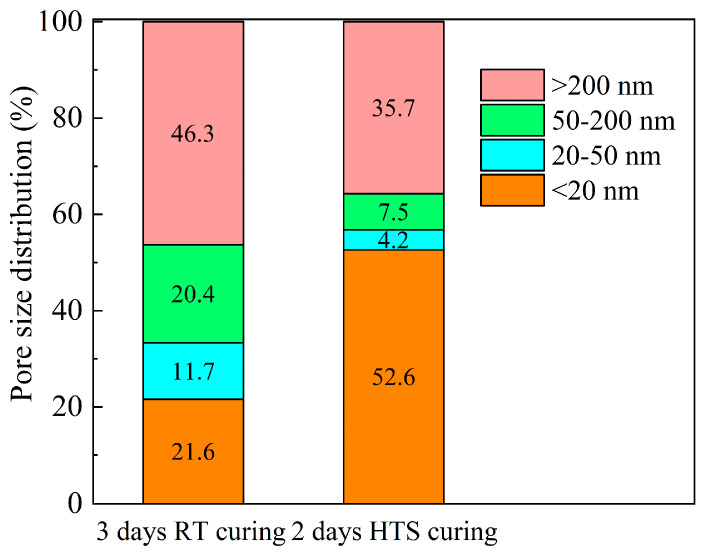
Statistics of pore size distribution of HPC−SW specimens cured by SC and HTS curing.

Pore structure analysis of HPC-SW specimens cured by different curing methods has been shown in Figure 4. Specifically, Figure 4a depicts the pore size distribution of HPC-SW specimens and the cumulative pore volume situation of the specimens are shown in Figure 4b. Moreover, the detailed statistics of the pore size distribution of the specimens cured by different curing methods are shown in Figure 4. Moreover, it can be seen from the curve of pore size distribution as shown in Figure 4a, one probable pore with a size of 12 nm has been found in the specimen cured by HTS curing; while the specimens subjected to SC exhibit three probable pores with sizes of 12 nm, 77 nm and 433 nm, respectively. Moreover, the total porosity of the specimens cured by SC and HTS curing are 10.9% and 10.2%, respectively. It can be seen the total porosities of HPC-SW specimens cured by different curing methods show no obvious difference. However, the pore size distribution shows significant differences between the specimens. Specifically, the pores of HPC-SW specimens prepared in this research are divided into four parts: the harmless pores of d < 20 nm; a little harmful pore with a pore size range of 20 < d < 50 nm; harmful pores of d = 50~200 nm and many harmful pores with d > 200 nm [14,15,16]. It can be observed from Figure 5 that many harmful pores account for a large proportion with the value of 46.3% in SC specimens. Moreover, the harmless pores take a small proportion (21.6%). The pore size distribution statistics indicate a poor pore structure inside the specimen cured by SC. However, HTS cured HPC-SW specimen exhibits a different pore size distribution situation. Specifically, the harmless pores account for a dominating role in the HTS cured specimen with the value of 52.6%, the total proportion of the little harmful pores and harmful pores in the specimen cured by HTS curing is equal to the proportion of the little harmful pores inside the SC specimen. This suggests the significant effect of HTS curing on refining the pore structure of HPC-SW specimens, and this is consistent with the mechanical properties as above.

### 3.5. Ion’s Content

**Figure 6:**
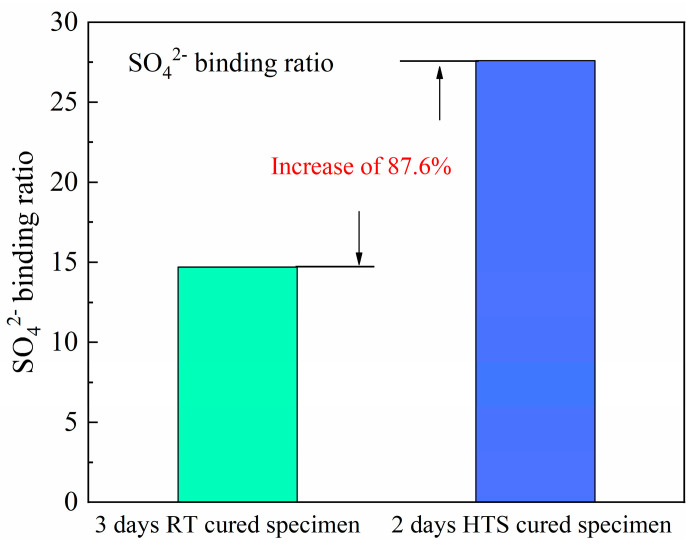
SO_4_^2−^ binding ratios of HPC−SW specimens cured by different curing regimes.

Free SO_4_^2−^ is the main harmful ion in the sludge water utilized in this work, and the existence of SO_4_^2−^ can lead to serious degradation issues inside the concrete structure. To be specific, the existence of SO_4_^2−^ can induce a complicated chemical reaction inside the specimen, leading to the formation of expansive phases including gypsum (CaSO_4_^−^·2H_2_O), thaumasite (Ca_3_[Si(OH)_6_·12H_2_O]·(CO_3_)·SO_4_), ettringite ([Ca_3_Al(OH)_6_·12H_2_O]_2_·(SO_4_)_3_·2H_2_O), or the mixtures of these products [17,18,19]. The formation of too many expansive phases may induce unavoidable cracks inside the specimen, which is harmful to concrete structures. Under this circumstance, the contents of SO_4_^2−^ are expected to be limited to a large extent to lower the risk of this harmful ion. The binding ratio of SO_4_^2−^ of HPC-SW specimens subjected to SC and HTS curing is shown in Figure 6. It can be seen from Figure 6 that the implementation of HTS curing can significantly increase the SO_4_^2−^ binding ratio of HPC-SW specimens. To be specific, the SO_4_^2−^ binding ratio of the specimens cured by SC is 14.7%; however, the HTS cured specimen exhibits a much stronger SO_4_^2−^ binding ratio of 27.6%, meeting an increase of 87.6% compared with that of the specimen cured by SC. Moreover, this is considered to be the reason that HTS curing is a curing method with higher curing temperature, such a high temperature can effectively improve the hydration degree and pozzolanic reaction inside the HPC specimens, which is beneficial for the ion immobilization inside cement-based materials.

## 4. Conclusions

In conclusion, sludge water has been incorporated in HPC specimens to substitute the fresh water in the HPC specimens. It has been found that the incorporation of SW shows no adverse effect on the mechanical properties of HPC specimens. The effect of different curing regimes on HPC-SW specimens are comprehensively studied. The main conclusions are listed as follows.

(1) The incorporation of SW shows no adverse effect on the mechanical properties of HPC specimens. Specifically, the compressive strength of the SC cured HPC-SW specimen reaches up to 85.2 MPa, showing little difference with that of the HTS cured HPC-FW specimen with the value of 84.3 MPa. Moreover, the flexural strength of HPC specimens exhibits a similar development regularity with that of compressive strength.

(2) Microstructural analyses indicate that the HTS curing can effectively improve the hydration degree and refine the pore structure of the HPC-SW specimens, indicating the priority for the combination of HTS curing and sludge water incorporation for green concrete structure construction.

(3) The results of SO_4_^2−^ binding ratio suggest the priority of HTS curing on improving the ion immobilization efficiency of HPC specimens, leading to the safe disposal of sludge water as mixing water for concrete construction.

## Figures and Tables

**Table 1:** Detailed composition of OPC in this work.

Materials	$SiO_{2}$	$Al_{2}O_{3}$	$Fe_{2}O_{3}$	MgO	CaO
Cement	20.86	5.47	3.94	1.73	62.23

## Data Availability

The data that support the plots within this paper and other findings of this study are available from the corresponding author upon reasonable request.
